# Determination of the Ni(II) Ions Sorption Mechanism on Dowex PSR2 and Dowex PSR3 Ion Exchangers Based on Spectroscopic Studies

**DOI:** 10.3390/ma16020644

**Published:** 2023-01-09

**Authors:** Justyna Bąk, Weronika Sofińska-Chmiel, Maria Gajewska, Paulina Malinowska, Dorota Kołodyńska

**Affiliations:** 1 Department of Inorganic Chemistry, Institute of Chemical Sciences, Faculty of Chemistry, Maria Curie-Skłodowska University, Maria Curie-Skłodowska Sq. 2, 20-031 Lublin, Poland; 2 Analytical Laboratory, Institute of Chemical Sciences, Faculty of Chemistry, Maria Curie Skłodowska University, Maria Curie Skłodowska Sq. 3, 20-031 Lublin, Poland

**Keywords:** XPS spectroscopy, FTIR spectroscopy, sorption, ion exchangers, Dowex, nickel ions

## Abstract

This paper estimates the suitability of the strongly basic anion exchangers, Dowex PSR2 and Dowex PSR3, as sorbents of nickel ions in aqueous solutions. These actions are aimed at searching for new solutions due to the growing discharge of nickel into wastewaters, primarily due to its addition to steel. The nickel sorption experiments were conducted under static conditions and resulted in the optimization of pH, phase contact time, initial solution concentration, and temperature. The next step was to calculate the kinetic, isothermal, and thermodynamic parameters. Moreover, the ion exchangers were characterized by means of Fourier transform infrared spectroscopy, X-ray photoelectron spectroscopy, and CHN elemental analysis. It was found that the sorption process was most effective at pH 6 after 240 min and at the temperature of 293 K. The values of the thermodynamic parameters revealed that the adsorption was exothermic and spontaneous. The physicochemical analyses combined with the experimental research enabled determination of the sorption mechanism of Ni(II) ions.

## 1. Introduction

**Figure 1:**
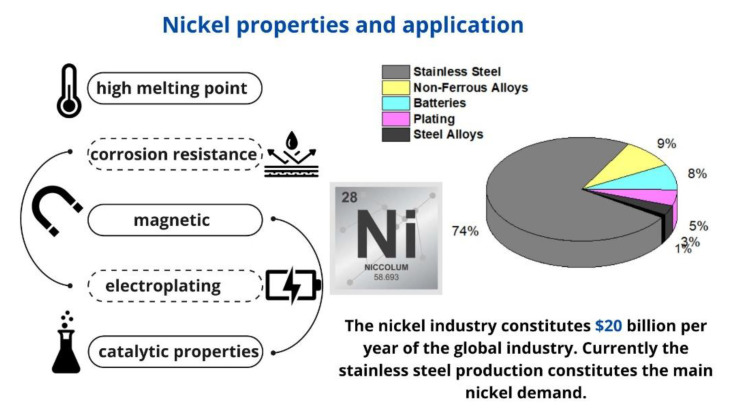
Nickel properties and application.

Economic growth in the world involves the use of numerous materials that are dangerous to the natural environment. As a result, the risk of air, water, and soil pollution is increasing [1,2]. The most dangerous pollutants for humans are heavy metals, poorly decomposing organic substances, and insoluble mineral compounds. An example of a heavy metal with allergic and carcinogenic effects is nickel [3,4,5]. The WHO recommends less than 0.1 mg/L of Ni(II) as the maximum permissible content in drinking water [6]. In terms of its distribution on earth, nickel ranks seventh. The average nickel content in the Earth’s crust is 100 mg/kg, and in the seawater it is about 5.4 g/L. Despite its wide distribution in nature, pure nickel itself is rare, most often in the form of sulfide, oxide, and silicate minerals [7]. Due to its properties, this element has many industrial applications. Nickel as a metal is malleable and suitable for welding, forging, or rolling. In addition, it forms alloys that are easily resistant to high temperatures and corrosion [8]. In addition, under the normal conditions, the metal is stable in water and air. Due to these properties, nickel is used in the electroplating industry to protect the surfaces of other metals [9]. This element is also used as a catalyst due to the fact that its crystal lattice can absorb hydrogen atoms [10]. The electrochemical properties of nickel are also used in the production of batteries, cadmium–nickel, and nickel–metal hydride cells [11,12]. This element is widely applied in the production of stainless steel. In the jewelry industry, despite its allergenic properties, nickel is used to make jewelry or eyeglass frames [13]. The basic information about nickel properties and applications is presented in Figure 1.

Ecology as a whole and the groundwater could be seriously endangered by nickel contamination from industrial and mining activities. This is dangerous due to the bioaccumulative nature of this element [14]. There are many ways to purify water, and one of the most appreciated is the ion exchange with the use of ion exchangers [15,16]. Compared with purification by precipitation or solvent extraction, the usage of ion exchange resins can offer a more efficient and straightforward purification [17]. Moreover, the ion exchange has also been successfully applied in a variety of applications, including the recovery of heavy metals from industrial wastes and the separation of rare earths or gas combinations to enhance the treatment of industrial effluents, as well as in the food sector [18]. There are many studies examining the efficiency of removing nickel ions from solutions with the use of various types of ion exchangers. Kołodyńska [19] described the results of research on the removal of Cu(II), Zn(II), and Ni(II) ion complexes with 1-hydroxyethylene-1,1-diphosphonic acid (HEDP) using Amberlite IRA 458, Amberlite IRA 67, Amberlite IRA 958, Purolite S-920, and Purolite S-930. Heavy metal complexes with HEDP within 20 min and at pH 11.5 are very well sorbed (even above 99%) on the highly basic anion exchangers Amberlite IRA 458 and Amberlite IRA 958, as well as the chelating ion exchangers Purolite S-920 and Purolite S-930, with the exception of the weakly basic anion exchanger Amberlite IRA 67, where the optimum pH was determined to be 7.0 [19]. Stefan and Meghea [20] investigated the simultaneous removal of Ca(II), Ni(II), Pb(II), and Al(III) ions using Purolite S-930. It was found that the ion exchange capacity of Ca(II), Ni(II), and Pb(II) ions increases relatively linearly in the pH range from 2.0 to 6.5. The greatest selectivity of the resin occurs for Ni(II), then Pb(II), while for the Al(III) ions, the maximum value of q_t_ is observed at pH 4.45. At higher pH values, the sorption capacity decreases due to the microprecipitation of aluminum on the resin grid [20]. Dizge et al. [21] conducted studies on the sorption of nickel(II) ions from the aqueous solution using Lewatit MonoPlus SP112 (strongly acidic, macroporous cation exchange resin). The adsorption process was relatively fast, and it took about 90 min to reach equilibrium. However, approximately 3 h of the contact time was required to achieve the process efficiency of 95–100%. The maximum adsorption capacity of the resin was 170.94 mg/g at 298 K according to the Langmuir isotherm [21].

**Figure 2:**
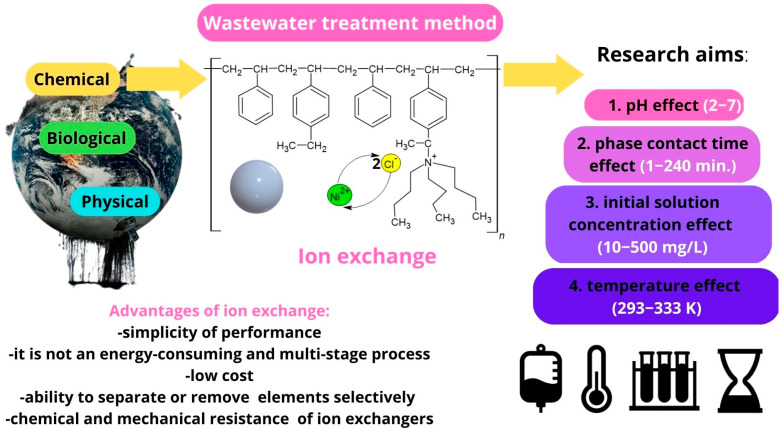
General scheme of ion exchange process and research objectives.

The purpose of this study was to evaluate the effectiveness of Ni(II) ion removal from aqueous solutions using the ion exchangers manufactured by DOW Chemical Company (Midland, MI, USA). In order to design technologies for the purification of water from heavy metals, it is necessary to research the structure and properties of materials, especially since there are very few literature reports on them. Understanding the structure of the exchangers combined with the results of research on the reaction rate and factors influencing the sorption processes (pH, phase contact time, initial concentration of the solution, and temperature) is undoubtedly a novel aspect of this paper that will allow us to fully understand the reaction mechanisms, as well as to determine the potential of the tested materials. Figure 2 depicts the general scheme of ion exchange, as well as the purpose of the research.

## 2. Materials and Methods

### 2.1. Sorbate and Sorbents Characteristics and Calculations

The appropriate amount of Ni(NO_3_)_2_∙6H_2_O (analytically pure) (Avator Performance Materials, Gliwice, Poland) was dissolved in distilled water and a 1000 mg/L nickel ions stock solution was prepared. Working solutions for the studies of the kinetics and sorption isotherms with the concentrations in the range of 10–500 mg/L were prepared by diluting the stock solution. The dropwise additions of 1 mol/L HNO_3_ and/or 1 mol/L NaOH to the solution promoted obtaining the required pH. A laboratory shaker (Elpin + 358A, Katowice, Poland) was used to shake the 100 mL Erlenmeyer flasks containing 0.1 g of ion exchanger and 50 mL of solution continuously at 180 rpm and the amplitude 8 during the batch adsorption studies. The impacts of pH (2–7), interaction time (1–240 min), initial adsorbate concentrations (10–500 mg/L), and temperature (293–333 K) were tested. After shaking, the sorbent was removed from the mixture by filtration, and the pH of the filtrate was assessed using the pHmeter pHM82 (Radiometer, Copenhagen, Denmark). After that, the atomic absorption analyzer SpectrAA240 FS (Varian, Palo Alto, CA, USA) at the wavelength of 232.0 nm was applied to determine the final nickel ions concentration. The basic adsorption parameters were calculated according to the equations:

(1) $$q_{t}=\frac{(C_{0}-C_{t})V}{m}$$

(2) $$q_{e}=\frac{(C_{0}-C_{e})V}{m}$$

where q_t_ and q_e_ are the adsorption capacities at time t (mg/g) and equilibrium (mg/g), respectively. C_0_, C_t_, and C_e_ are the initial, after time t, and at the equilibrium concentrations (mg/L), respectively, V is the solution volume (mL), and m is the sorbent mass (g).

The physicochemical properties of Dowex PSR2 and Dowex PSR3 are listed in Table 1.

The kinetic models, such as the pseudo-first-order (PFO), pseudo-second-order (PSO), Elovich kinetic equations (EKE), and intraparticle diffusion (IPD) ones, were applied to evaluate the results. To analyze the equilibrium data, the Langmuir, Freundlich, Temkin, and Dubinin–Raduszkiewicz, as well as Sips, isotherm models were used. The formulae and parameters of the kinetic and isotherm models are presented in Table 2.

Using the formulae provided in [22], the thermodynamic parameters’ entropy change ΔS° (kJ/mol), free energy change ΔG° (kJ/mol), and enthalpy change ΔH° (kJ/mol) were calculated and interpreted.

### 2.2. FTIR Spectroscopy

The FTIR-ATR spectroscopic experiments were conducted to study the chemical composition and clarify the mechanism of Ni(II) ions sorption on Dowex PSR2 and PSR3. The FTIR Thermo Nicolet 8700 (Thermo Scientific, Waltham, MA, USA) spectrometer with the Smart OrbitTM attachment diamond ATR and the DTGS (deuterated triclycine sulfate) detector was used to test the Dowex PSR2 and Dowex PSR3 ion exchanger samples before and after the Ni(II) ions sorption process at 10, 100, 200, and 500 mg/L. Before the measurements, the samples of ion exchangers were ground in the agate mortar. The tests were carried out at room temperature in the wavenumber range of 4000–400 cm^−1^, with the spectral resolution of 4 cm^−1^. A total of 64 scans were made for each spectrum. Using the Omnic SpectaTM software(number 833-036200), the collected spectra were subjected to ATR correction, automated baseline correction, and normalizing.

### 2.3. XPS Spectroscopy

In order to examine the chemical structure and explain the mechanism of Ni(II) ions sorption on Dowex PSR2 and Dowex PSR3, the XPS tests were made using the Ultra-High Vacuum multichamber analytical system (Prevac, Rogów, Polska). The tests were carried out using Dowex PSR2 and Dowex PSR3 before and after the Ni(II) ions sorption. The samples were degassed at ambient temperature to a high continuous vacuum of approximately 5 × 10^−8^ mbar in the UHV system sluice after being fixed on a molybdenum carrier. Following their entrance into the system’s analytical chamber, the proper analysis was carried out using the XPS spectroscopy method. Spectra were collected using the hemispherical Scienta R4000 electron analyzer (Scienta Scientific AB, Uppsala, Sweden) The Scienta SAX-100 X-ray source (Al Kα, 1486.6 eV, 0.8 eV band) equipped with the XM 650 X-ray Monochromator (Inrad Optics Inc., Northvale, NJ, USA) (0.2 eV band) was used as a complementary.

The pass energy of the analyzer was set to 200 eV for the survey spectra (with 500 meV step) and 50 eV for the C1s region (high-resolution spectra with the 50 meV step). The base pressure in the analysis chamber was 5 × 10^−9^ mbar. During the spectra collection, it was not higher than 3 × 10^−8^ mbar.

### 2.4. CHN Analysis

The samples of Dowex PSR2 and Dowex PSR3 ion exchangers before the sorption process were tested using the CHN/CHNS EuroEA3000 analyzer (EuroVector, Milan, Italy) which enables automatic and simultaneous determination of the percentage content of carbon, hydrogen, and nitrogen. Prior to the measurements, the samples were weighed using the analytical microbalance M2P by Sartorius, with the accuracy of 0.001 mg. The test was based on the Dumas dynamic combustion method, followed by the chromatographic separation of gaseous fractions resulting from this combustion (N_2_, CO_2_, and H_2_O), and then their analysis using the catarometer. The test results were compiled using the Callidus package. Two combustion processes were performed for each sample.

## 3. Results and Discussion

### 3.1. Optimization of the Sorption Process

**Figure 3:**
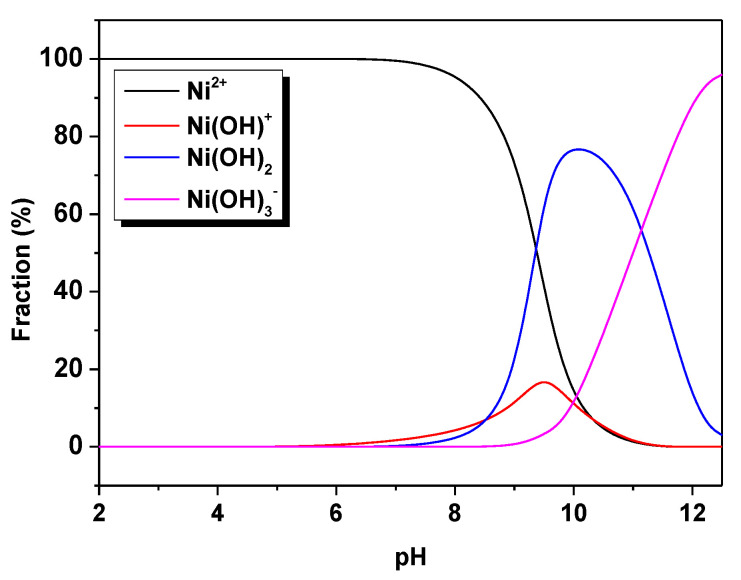
Speciation diagram of nickel.

Based on the nickel speciation plot, it is concluded that pH plays an important role in the sorption process. Figure 3 indicates the prevalent Ni species at various pH values, for example, Ni^2+^ at pH < 8.0, Ni(OH)^+^ at pH 9.0–10.0, Ni(OH)_2_ at pH 10.0–11.0, and Ni(OH)_3_^−^ at pH > 11.0. At pH > 6.7, the majority of nickel exists in the form of solid hydroxides, but pH 6.5 is found to make all nickel complexes generally soluble [23].

**Figure 4:**
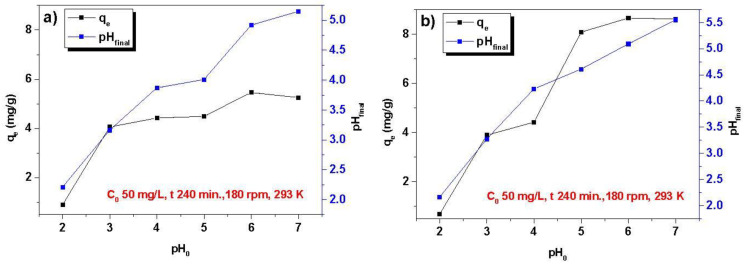
Impact of solution pH on the nickel ions sorption on (**a**) Dowex PSR2 and (**b**) Dowex PSR3.

Figure 4 presents the effects of Ni(III) ions sorption on Dowex PSR2 and PSR3. The research was carried out in the pH range of 2.0–7.0, because at pH above 7.0 nickel hydroxide precipitated. The amount of adsorbed ions increases gradually as pH rises from 2.0 to 6.0, and the maximum value is obtained at pH 6.0. As a result, this amount was chosen for further investigations. The amount of adsorbed Ni(II) ions was 5.46 mg/g for PSR2 and 8.65 mg/g for PSR3. Furthermore, the final pH value increases after the sorption process.

**Figure 5:**
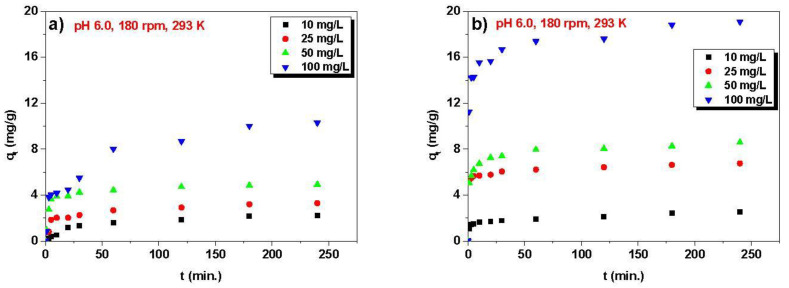
Impact of the phase contact time and the initial concentration of the solution on the nickel ions sorption on (**a**) Dowex PSR2 and (**b**) Dowex PSR3.

The next step in optimizing the sorption process was determining the effects of phase contact time and initial concentration of solution (Figure 5). The studies were carried out in the contact time range of 0–240 min and the initial solution concentration from 10 to 100 mg/L.

The value of adsorbed Ni(II) ions increases with time, regardless of the initial solution concentration, which improved adsorption efficiency. After 240 min, the q_t_ values were equal to: 2.24, 3.30, 4.92, and 10.30 mg/g for the concentrations: 10, 25, 50, and 100 mg/L, respectively, for Dowex PSR2. These values for Dowex PSR3 for the same concentrations are: 2.51, 6.76, 8.59, and 19.10 mg/g. The much greater amounts of the adsorbed ions, especially for the concentration of 100 mg/L for the PSR3 ion exchanger, indicate a greater affinity of nickel ions for PSR3. The same results were obtained by Wołowicz [24], who investigated the zinc(II) ions removal from the model chloride and chloride–nitrate(V) solutions using different ion exchangers, among others, PSR2 and PSR3. In the zinc(II) sorption from the chloride solution at 0.1 mol/L concentration, the q_t_ equals 2.4 mg/g for PSR2 and 4.1 mg/g for PSR3 [24]. Additionally, it was found that the initial solution concentrations affected the time required to reach the equilibrium of sorption. A total of 30 min of sorption time is sufficient to reach the equilibrium for the concentration of 10 mg/L. For 25 and 50 mg/L, the time is extended to 60 min. The slowest equilibration occurs at the highest concentration of 100 mg/L.

Table 3 shows the comparison of the equilibrium capacities and sorption conditions of the other ion exchangers in relation to the nickel ions. Many authors have undertaken research on the removal of nickel ions from aqueous, chloride, and nitrate solutions under various conditions. On the basis of the equilibrium capacities listed in the table, it can be concluded that Dowex PSR2 and PSR3 have high q_e_ values. Wołowicz and Hubicki [25] carried out research under similar conditions (C_0_ 100 mg/L, t 240 min, and T 298 K), and the only difference was the removal of nickel ions from the chloride solutions at different concentrations of HCl, from 0.1 to 6.0 mol/L. Regardless of the hydrochloric acid concentration, smaller amounts of adsorbed Ni(II) ions were obtained. Therefore, it can be stated that the removal of nickel ions from the aqueous solutions using the Dowex PSR2 and PSR3 ion exchangers is very effective and can be successfully transferred to an industrial scale.

**Figure 6:**
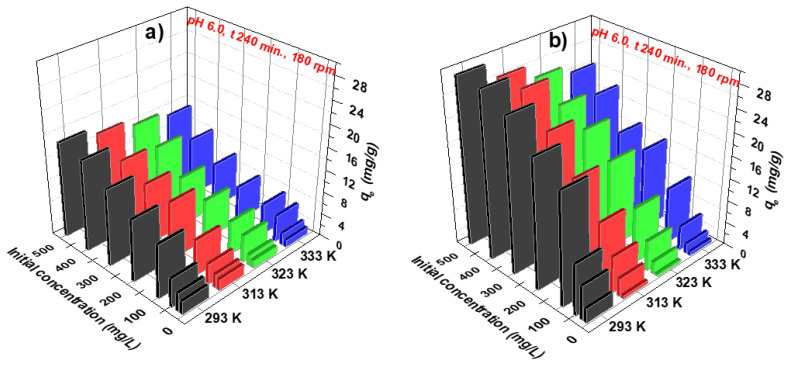
Impact of temperature on the nickel ions sorption on (**a**) Dowex PSR2 and (**b**) Dowex PSR3.

The sorption process was optimized studying the impact of temperature in the next step. In this case, the investigations were carried out at 293, 313, 323, and 333 K for the initial nickel ions concentrations from 10 to 500 mg/L (Figure 6). These tests will also be used to calculate the thermodynamic parameters of the process and to indicate its endo- or exothermic character.

The equilibrium capacity increases with the increasing initial concentration, reaching 16.93 mg/g at the highest concentration for Dowex PSR2 and a temperature of 293 K. As the temperature increases, it can be seen that the q_e_ values decrease and are equal to 15.51, 14.14, and 13.09 mg/g for 313, 323, and 333 K, respectively (for the concentration of 500 mg/L and PSR2). For Dowex PSR3, the q_e_ values changed as follows: 29.40, 26.59, 24.09, and 22.48 mg/g for the temperatures of 293, 313, 323, and 333 K. The decrease in the nickel ion diffusion toward the outer surface and into the pores of ion exchangers caused by the rise in temperature can be utilized to explain this. Thus, the optimal temperature for nickel ions sorption on Dowex PSR2 and PSR3 is 293 K. These investigations also demonstrate the greater efficiency of removing nickel ions from the aqueous solutions using Dowex PSR3.

### 3.2. Kinetic, Isotherm, and Thermodynamic Parameters

As can be seen in Table 4 and Table S1, the parameters of the two kinetic models showed the better fit of the PSO model than that of the PFO one with higher R^2^ values. Additionally, the q_e_ values predicted by the PSO model were in excellent agreement with the experimental values. This suggests that this model was more appropriate to describe the sorption behavior of Ni(II) ions. This can reflect the fact that the nickel removal is controlled by both surface diffusion and chemical reactions at the liquid–solid interface [34].

The PSO model, which does not take into account the reaction mechanism, exhibits a fit for the whole time period under study. Due to the fact that the PFO and PSO models do not describe the adsorptive diffusion mechanism, the intraparticle diffusion (IPD) model is used for this purpose. This is to define the steps that control the rate of the reaction [35]. The first stage is related to the diffusion of heavy metal ions through the solution to the outer sorbent surface. In this stage, the adsorption process is fast, and the curve is steeply sloped. The intramolecular diffusion process slows down the reaction rate in the second step, flattening the curve as a result. In the third stage, when the concentration of ions in the solution decreases due to reaching the equilibrium, the diffusion process slows down [36]. The values of the coefficients k_i_, varying in the range k_i1_ > k_i2_ > k_i3_, suggest a reduction in the reaction rate for the subsequent stages of the diffusion process.

The Elovich equation (EKE) is used to describe chemisorption. The increase in the initial Ni(II) ions concentration from 10 to 100 mg/L resulted only in a slight decrease in the desorption constant (β) for Dowex PSR2 from 2.190 to 0.562 g/mg and from 3.885 to 0.735 g/mg for Dowex PSR3. A small β value (<1) suggests an irreversible adsorption process. Interestingly, for the concentrations in the range of 10–50 mg/L, the β values are above unity, suggesting reversibility of the process, and for the concentrations of 100 mg/L, they are below unity, which may suggest an irreversible process [37]. For the EKE model, obtained correlation coefficients are greater than 0.909. It should be noted that the other kinetic models provide a superior fit with the experimental data, notwithstanding the Elovich equation utility in estimating reaction kinetics.

Table 5 displays the findings of the analysis of the equilibrium data using the Langmuir, Freundlich, Temkin, and Dubinin–Raduszkiewicz isotherms. The assumption that the uniform adsorption takes place on the sorbent active sites forms the basis of the Langmuir isotherm, which was used to characterize the adsorption phenomena. Furthermore, once an adsorbate occupies an active site, no more sorption can take place there. On the other hand, the nonideal sorption on the heterogeneous surfaces was described by means of the Freundlich isotherm model [38,39]. Compared with the determination coefficients of the Langmuir and Freundlich models, higher values were obtained for the Langmuir one. The maximum removal of Ni(II) ions predicted by the Langmuir model was 19.16 mg/g for Dowex PSR2 and 31.72 mg/g for PSR3. These values are slightly overestimated, because the experimental data equal 16.93 mg/g for PSR2 and 29.40 mg/g for PSR3.

According to the Temkin model, the heat of adsorption for all particles decreases linearly with an increase in the surface area covered by the adsorbent. Additionally, the spread of binding energy up to the topmost binding energy describes the adsorption process precisely [40]. The change in the adsorption energy is associated with the b_T_ constant of the Temkin isotherm. An endothermic adsorption reaction is indicated by a negative value of the b_T_ constant. However, in this research, the exothermic nature of the adsorption reaction is suggested by the found positive values, which is supported by the thermodynamic parameters [41]. The calculated determination coefficient ≥0.915 indicates the participation of electrostatic interactions in the sorption process.

The Dubinin–Raduszkiewicz isotherm model defines the heterogeneity of ion adsorption on the surface and makes a distinction between physical and chemical adsorption processes [40]. The evaluation of the sorption character is based on the estimated parameter E_a_ value, which is defined as the free energy transfer of 1 mole of the solute from the infinite of the sorbent surface [42]. When E_a_ is in the range of 1–8 kJ/mol, it indicates physisorption. The activation energy from 8 to 16 kJ/mol indicates the ion exchange. On the other hand, the values in the range of 16–40 kJ/mol confirm chemisorption [42,43]. The activation energies: 10.074 kJ/mol for Dowex PSR2 and 9.753 kJ/mol for PSR3 typify ion exchange as the main mechanism for removing nickel ions.

The Sips isotherm combines the Freundlich and Langmuir isotherms and is useful in heterogeneous adsorption, in which the adsorbed molecule has several adsorption sites. Adsorbate–adsorbate synergy is not taken into account by the model. The estimated adsorption capacity for PSR2 is overpriced and equals 27.74, but for PSR3, it is understated and equals 25.53 mg/g. For Dowex PSR2, the correlation coefficient results were best fitted with the Sips isotherm model with R^2^ ≥0.982.

Table 6 lists the thermodynamic parameters of Ni(II) ions sorption on two ion exchangers. The negative value of ΔH° suggests that the process is exothermic, which is confirmed by the values of b_T_ parameters determined on the basis of the Temkin isotherm. The adsorption process is spontaneous, as indicated by the positive value of ΔS° and the negative value of Gibbs free energy (ΔG°). Additionally, the values of the ΔG° decrease with rising temperature, which proves that the sorption process is more effective at lower temperatures [44,45].

### 3.3. Physicochemical Analyses

#### 3.3.1. FTIR Spectroscopy

**Figure 7:**
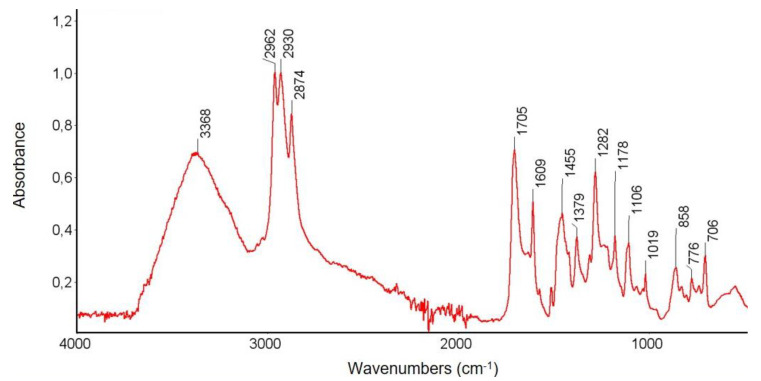
FTIR-ATR spectrum of Dowex PSR2 ion exchanger sample before the sorption process.

**Figure 8:**
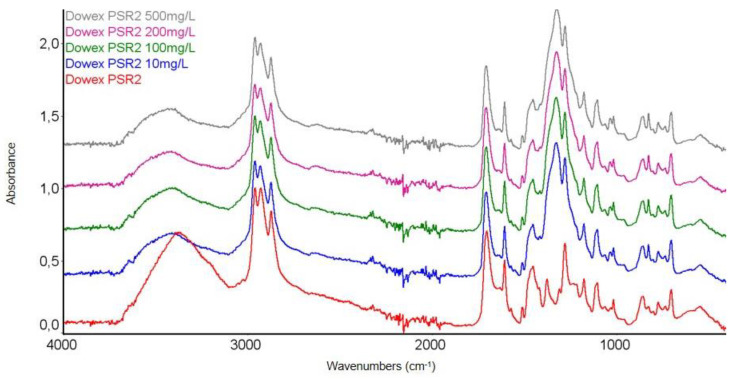
Summary of the FTIR-ATR spectra of the Dowex PSR2 ion exchanger samples before and after the Ni(II) ions sorption process at 10, 100, 200, and 500 mg/L.

**Figure 9:**
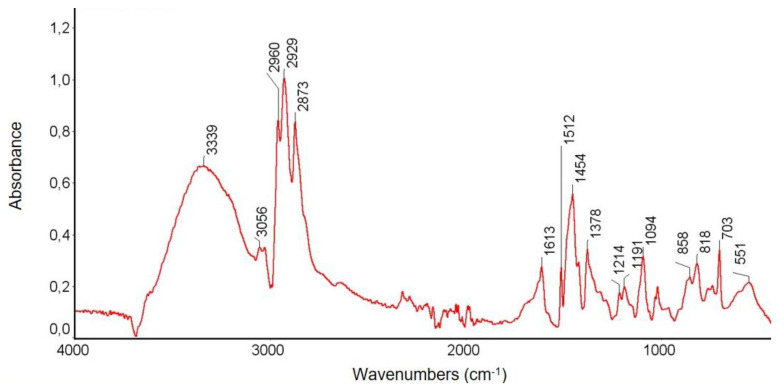
FTIR-ATR spectrum of Dowex PSR3 ion exchanger sample before the sorption process.

**Figure 10:**
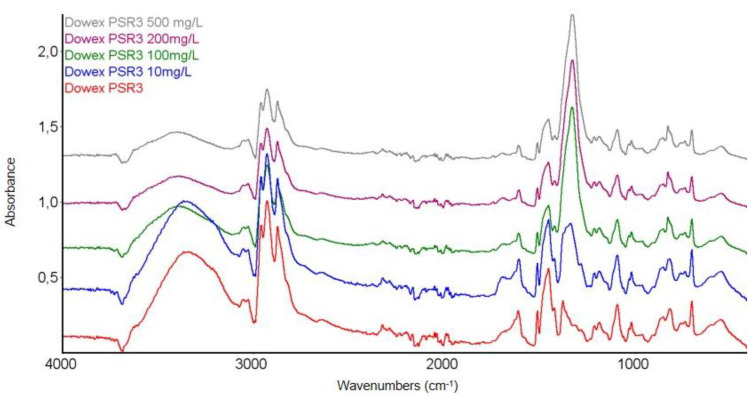
Summary of the FTIR-ATR spectra of the Dowex PSR3 ion exchanger samples before and after the Ni(II) ions sorption process at 10, 100, 200, and 500 mg/L.

In order to confirm the chemical structure of the tested Dowex PSR2 and Dowex PSR3 ion exchangers and to observe the changes occurring under the influence of Ni(II) ions sorption, FTIR spectroscopic studies were carried out using the ATR technique. FTIR-ATR spectra of the pure ion exchanger were obtained before the sorption process and after the Ni(II) ions sorption process, with the concentrations of 10, 100, 200, and 500 mg/L. The test results are presented in Figure 7, Figure 8, Figure 9 and Figure 10.

The FTIR-ATR tests of the Dowex PSR2 ion exchanger before the sorption process showed a band of great intensity in the range of 3600–3200 cm^−1^. The stretching vibrations of the O-H groups and the N-H groups are represented by this band [46]. In the wavenumber range of 3090–3000 cm^−1^, the presence of bands corresponding to the stretching vibrations of the C-H groups was found [47]. The FTIR-ATR study also presented the presence of a band corresponding to the symmetric and asymmetric stretching vibrations originating from the aliphatic CH_2_ groups in the wavenumber range of 3000–2850 cm^−1^ [46,47,48]. An intense peak at 1705 cm^−1^ was also found, corresponding to the stretching vibrations of the C=O groups [49]. In the wavenumber range of 1610–1500 cm^−1^, the bands’ characteristic of symmetric C=C stretching vibrations of aromatic rings, characteristic of the ion exchange matrix, at 1454, 1378, and 900–703 cm^−1^, were observed [29,50,51]. The presence of numerous, low-intensity bands, including deformation vibrations of CH_2_ groups, as well as stretching vibrations of C-C in the range of 1215–1150 cm^−1^, were found. The tests also showed the presence of delocalized skeletal vibrations of the ion exchanger matrix in the position of 552 cm^−1^.

The FTIR spectroscopic studies carried out on the Dowex PSR2 ion exchanger after the Ni(II) ions sorption process showed the presence of the same FTIR-ATR spectral bands as found before the sorption process. However, some differences in signal intensity across the spectrum were observed. The intensity of the signals corresponding to the stretching vibrations of the O-H and the N-H groups (in the range of 3600–3200 cm^−1^) decreases as the concentration of the Ni(II) ions solution rises. With the increasing concentration of Ni(II) ions in the FTIR spectra, changes in the peak intensity in the range of 1530–1250 cm^−1^ were observed. In this range, there are peaks in positions 1512 cm^−1^, 1454, 1378, and 900–703 cm^−1^, corresponding to the stretching vibrations of aliphatic amino groups present in the ion exchanger functional groups [29,50,51]. Changes in the intensity of the peaks in this range may indicate the ongoing sorption process involving the nitrogen atoms present in the tri-n-butylammonium functional groups.

In the FTIR-ATR spectra of the Dowex PSR3 ion exchanger, a band of high intensity in the range of 3600–3200 cm^−1^ was observed before the sorption process. This band corresponds to the stretching vibrations of the O-H groups and the N-H groups [52]. At the wavenumbers in the range of 3090–3000 cm^−1^, stretching vibrations of the C-H groups were observed [47]. In the range of 3000–2850 cm^−1^, the bands corresponding to the symmetric and asymmetric stretching vibrations originating from the aliphatic CH_2_ groups were observed [46,47,48]. In the wavenumber range of 1610–1500 cm^−1^, the bands characteristic of the symmetric C=C stretching vibrations of the aromatic rings of the ion exchanger matrix were observed. The tests showed the presence of stretching vibrations originating from the aliphatic amino groups at the following signal positions: 1512 cm^−1^, 1454, 1378, and 900–703 cm^−1^ [29,50,51]. The FTIR spectra also showed the presence of numerous, low-intensity bands covering the deformation vibrations of the CH_2_ groups, and stretching C-C in the range of 1215–1150 cm^−1^. In the position of 552 cm^−1^, the presence of delocalized skeletal vibrations of the ion exchanger matrix was found.

Comparison of the FTIR spectra of the Dowex PSR3 ion exchanger before and after the process of Ni(II) ions sorption also showed significant differences in the signal positions in the range of 1454–1200 cm^−1^. In this range, the signals corresponding to the stretching vibrations of the aliphatic amino groups present in the functional group of the ion exchanger were located. This may indicate the ongoing sorption process with the participation of nitrogen atoms present in the tri-n butylammonium functional groups [29,50,51].

#### 3.3.2. XPS Spectroscopy

**Figure 11:**
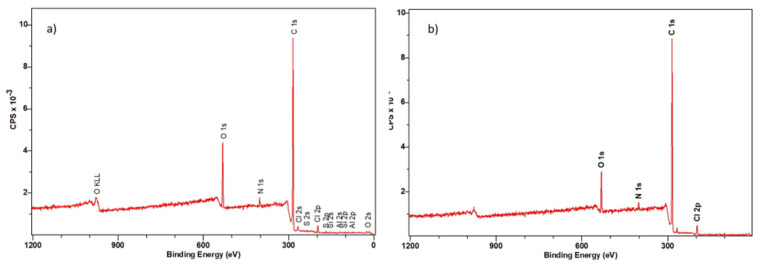
XRS spectra in a wide range of ion exchanger binding energies: (**a**) Dowex PSR 2 and (**b**) Dowex PSR3 before the process of Ni(II) ions sorption.

**Figure 12:**
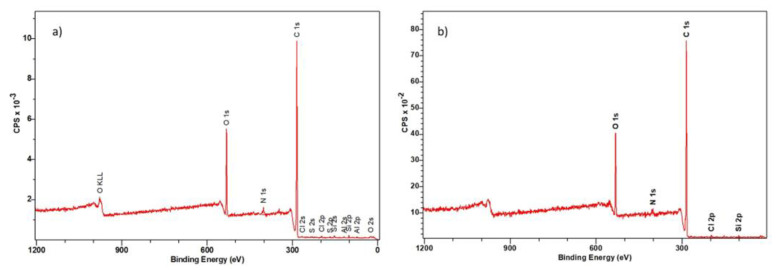
XRS spectra in a wide range of ion exchanger binding energies: (**a**) Dowex PSR 2 and (**b**) Dowex PSR3 after the Ni(II) ions sorption process.

To identify the elemental composition and chemical bonds characteristic of the tested ion exchanger, XPS spectroscopic tests were carried out. The analyses were made for the pure Dowex PSR2 and Dowex PSR3 ion exchangers and after the Ni(II) ions sorption with the concentration of 100 mg/L. The test results are given in Figure 11 and Figure 12 and Table 7, Table 8, Table 9 and Table 10.

The XPS tests of Dowex PSR2 and Dowex PSR3 ion exchangers before the Ni(II) ions sorption process showed the following elemental composition: Dowex PSR 2: 83.3% carbon, 2.2% nitrogen, 11.5% oxygen, and 1.6% chlorine; Dowex PSR3: 87.7% carbon, 1.6% nitrogen, 8.1% oxygen, and 2.7% chlorine. In addition, in the sample of the Dowex PSR 2 ion exchanger, the presence of aluminum, silicon, and sulfur was detected in an amount not exceeding 1 atomic %.

The XPS studies of Dowex PSR2 and Dowex PSR3 ion exchangers carried out in a narrow range of carbon binding energy showed the presence of bonds characteristic of both the ion exchange matrix (DVB cross-linked polystyrene) and tri-n-butylamine functional groups. The presence of C=C, C-H, C-C, and CN bonds and carbon bound to the aromatic groups was confirmed. The XPS tests carried out for oxygen showed the presence of nitrates and C-O bonds. The presence of chlorine in the form of Cl^−^ and chlorates ions was also observed. The presence of chlorates was confirmed in the Dowex PSR3 ion exchanger sample. The analyses of the Dowex PSR2 and Dowex PSR3 ion exchangers made in a narrow range of binding energy for nitrogen showed the presence of nitrogen in the form of a quaternary amine, which agrees with the information provided by the ion exchanger manufacturer. The presence of a protonated amine and nitro group was also observed in the Dowex PSR2 ion exchanger.

The XPS analysis of the Dowex PSR2 and Dowex PSR3 ion exchangers after the Ni(II) ions sorption process showed the following elemental compositions: Dowex PSR2: 80.10% carbon, 3.0% nitrogen, 14.4% oxygen, and 0.3% chlorine; Dowex PSR3: 82.3% carbon, 3.1% nitrogen, 14.2% oxygen, and 0.5% chlorine. In addition, the Dowex PSR2 ion exchanger sample contained aluminum, silicon, and sulfur in an amount not exceeding 1%. Due to the too low sensitivity of the method, the XPS tests for both ion exchangers after the Ni(II) ions sorption process did not show the presence of this element. The nickel content in the tested samples was determined by the XRF (X-ray fluorescence spectroscopy). The test showed a nickel content of 4.0 mg/L in the Dowex PSR2 ion exchanger and 6.4 mg/L in the Dowex PSR3 ion exchanger. The tests of ion exchangers carried out in a narrow range of binding energies for carbon showed the presence of the following bonds: C=C, C-H, C-C, CN, -COOH, and C-O and carbon linked to the aromatic groups. Particular attention in the XPS analysis was paid to the spectra of nitrogen after the Ni(II) ions sorption process. The XPS studies of the Dowex PSR2 ion exchanger after the sorption process showed a smaller number of groups in the form of quaternary amine from 31.5% to 21.1% and the protonated amine from 61.9% to 50.7%, which suggests the participation of nitrogen from the functional groups of the ion exchanger in the Ni(II) ions sorption process. In the case of the Dowex PSR3 ion exchanger, changes in the nitrogen spectra were also observed. A decrease in the amount of nitrogen was found in the form of quaternary amine from 100% to 66.8%, as well as the appearance of a nitro group which, as in the case of the Dowex PSR2 ion exchanger, indicates the participation of nitrogen in the Ni(II) ions sorption. The atomic percent is used to express the test results. In addition, the spectra of PSR2 and PSR3 before and after the sorption process in a narrow range of binding energies for C, O, N and Cl are presented in Supplementary Materials in Figures S1–S4.

#### 3.3.3. Elemental Analysis of CHN

Due to the fact that the hydrogen content is not determined by the XPS electron spectroscopy, the CHN analysis was performed for the pure ion exchangers. The test results are presented in Table 11.

To estimate the number of functional groups per one cross-linked polystyrene DVB, the theoretical calculations were made of the % content of elements included in the ion exchanger (carbon, nitrogen, and hydrogen). The calculation results are given in the mass %. Calculations were made for the following matrix:functional group configurations: 1:1, 2:1, and 1:2, and they are presented in Table 12.

The ratio of nitrogen present in the functional groups to carbon and hydrogen indicates the internal structure of the ion exchanger, in which one functional group occurs per two units of DVB cross-linked polystyrene.

### 3.4. Sorption Mechanism

The physicochemical properties of the Dowex PSR2 and Dowex PSR3 ion exchangers were examined before and after the sorption process to indicate the possible mechanism of Ni(II) ions sorption. The comparison of the FTIR spectra of the ion exchangers before and after sorption process revealed differences in the positions and intensities of spectral bands, which corresponded to the stretching vibrations of the aliphatic amino groups present in the functional groups of the ion exchanger. This shows how the boundaries of these groups change as a result of the Ni(II) ions sorption process.

The XPS spectroscopic studies on nitrogen after the sorption of Ni(II) ions revealed changes within the functional groups as a result of the sorption process. These changes imply that nitrogen from the quaternary amine is involved in the Ni(II) ions sorption process. Both ion exchangers showed a decrease in the amount of nitrogen in the form of the quaternary amine and an increase in the amount of NCl- bonds. The XPS studies showed a greater number of NCl-groups after the sorption process in the Dowex PSR3 ion exchanger compared with the Dowex PSR2 ion one. These studies corroborate the research conducted on the kinetics of the sorption process. In the case of the Dowex PSR3 ion exchanger, greater equilibrium capacities were obtained compared with the Dowex PSR2 ion exchanger. The results of XRF tests also confirm the greater efficiency of the Ni(II) ions sorption process on the Dowex PSR3 ion exchanger. The test showed a nickel content of 4.0 mg/L in the Dowex PSR2 ion exchanger and 6.4 mg/L in the Dowex PSR3 one.

Based on the parameters determined from the Temkin model and the thermodynamic parameters, it was found that the process is exothermic. In addition, the calculated activation energy from the Dubinin–Raduszkiewicz model confirms that ion exchange is the main mechanism of sorption.

As follows from the results of XPS tests for nitrogen, it can be assumed that, in the case of the Dowex PSR2 ion exchanger, one in four functional groups participates in the Ni(II) ions sorption process, and in the case of the Dowex PSR3 ion exchanger, one in three functional groups participates in the Ni(II) ions sorption process.

**Figure 13:**
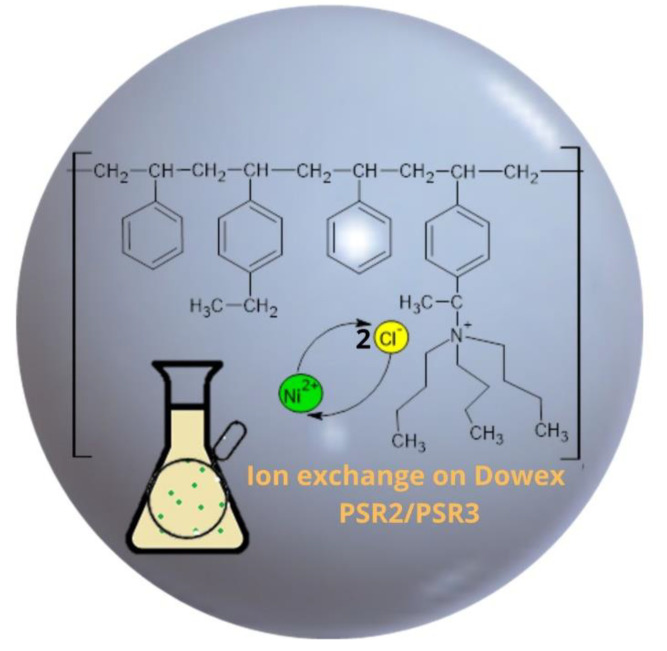
Proposed mechanism of the Ni(II) ions sorption process on the ion exchangers: Dowex PSR2 and PSR3.

The sorption studies, combined with the ion exchangers instrumental studies, allow us to propose the following mechanism for the Ni(II) ions sorption reaction on the Dowex PSR2 and Dowex PSR3 ion exchangers (Figure 13).

The figure shows that the sorption process takes place through the exchange of chloride ions present in the quaternary amine for nickel ions in stoichiometric terms—two NCl^−^ groups for one nickel ion.

## 4. Conclusions

The focus of this paper was an evaluation of the suitability of the strongly basic anion exchangers, Dowex PSR2 and Dowex PSR3, as potential sorbents in the removal of nickel ions from aqueous solutions. It was reported that pH 6 and 293 K are the ideal conditions for the sorption process. The phase contact time of 240 min is appropriate for establishing equilibrium, and the amount of adsorbed Ni(II) ions after this time is 10.30 mg/g for Dowex PSR2 and 19.10 mg/g for Dowex PSR3 (for the initial solution concentration of 100 mg/L). The PSO kinetic model showed the best fit of the experimental data to the theoretical ones and coefficients of determination close to unity. Based on the identified isotherms, it can be stated that in the case of the ion–exchangers–nickel ions solution systems, the best fit was observed on the Sips adsorption isotherm, where the R^2^ equals 0.982 for PSR2 and for PSR3, and the Langmuir isotherm, where R^2^ is equal 0.995. On the other hand, the parameters calculated from the Temkin isotherm model prove the exothermic nature of the process (which is confirmed by the enthalpy value) and indicate a share of electrostatic interactions in the process of removing nickel ions. The FTIR spectroscopic studies of the Dowex PSR2 and Dowex PSR3 ion exchangers showed the presence of bands characteristic of the ion exchanger matrix and tri-n-butylamine functional groups. The XPS spectroscopic studies allowed to determine the elemental composition of the tested ion exchangers and confirmed the presence of bonds in the functional groups and the ion exchanger matrix. The elemental composition tests carried out by means of the CHN elemental analysis method suggest the structure of the ion exchangers, in which there is one tri-n-butylammonium functional group for two polymers constituting the ion exchange matrix. The differences in the position and intensity of the spectral bands associated with the stretching vibrations of the aliphatic amino groups present in the functional groups of the ion exchanger indicate that chloride ions are exchanged for nickel ions within these groups. The dominance of ion exchange in the sorption process was confirmed by the activation energy calculated from the Dubinin–Raduszkiewicz model.

## Figures and Tables

**Table 1:** Comparison of physicochemical properties of the ion exchangers.

Features	Ion Exchangers	
	Dowex PSR2	Dowex PSR3
Resin type	strongly basic anion exchangers	strongly basic anion exchangers
Physical form	beige beads	beige beads
Skeleton	microporous, cross-linked polystyrene DVB	macroporous, cross-linked polystyrene DVB
Functional groups Matrix	tri-n-butyl ammonium 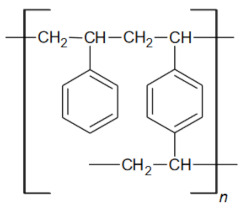	tri-n-butyl ammonium 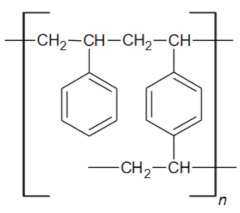
Ion exchange capacity (val/L)	0.65	0.60
Water retention (%)	40–48	50–65
Mean bead size (mm)	0.3–1.2	0.3–1.2
Working temperature (K)	<373	<373
Producer	DOW Chemical Company	DOW Chemical Company

**Table 2:** Kinetic and isotherm models.

Model	Equation	Parameters
Kinetic		
PFO	$\log(q_{1}-q_{t})=\log(q_{1})-\frac{k_{1}t}{2.303}$	k_1_—the rate constant of PFO equation (1/min)
PSO	$\frac{t}{q_{t}}=\frac{1}{k_{2}q_{2}^{2}}+\frac{t}{q_{2}}$	k_2_—the rate constant of PSO equation (g/mg∙min)
EKE	$q_{t}=\frac{1}{\beta }\ln(\alpha \cdot \beta )+\frac{1}{\beta }\ln(t)$	α—the initial adsorption rate (mg/g∙min) β—the constant of desorption connected with the activaton energy chemisorption and the range of surface coverage (g/mg)
IPD	qt=kit12+C	k_i_—the rate constant of intraparticle diffusion equation (mg/g/min^0.5^) C—the intercept which reflects the boundary layer impact
Isotherm		
Langmuir	$q_{e}=\frac{q_{0}K_{L}C_{e}}{1+K_{L}C_{e}}$	q_0_—the Langmuir monolayer sorption capacity (mg/g) K_L_—the characteristics of Langmuir equation (L/mg)
Freundlich	qe=KFCe1n	K_F_—the adsorption capacity of the Freundlich equation (mg/g) n—the Freundlich constant associated with the surface heterogenity
Temkin	$q_{e}=\frac{RT}{b_{T}}\ln(A\cdot C_{e})$	b_T_—the Temkin constant related to the sorption heat (kJ/mol) A—the equilibrium Temkin binding constant (L/g)
Dubinin-Raduszkiewicz	$\ln q_{e}=\ln q_{m}-\beta \varepsilon^{2}$ $E_{a}=\frac{1}{\sqrt{2\beta }}$	q_m_—the Dubinin–Raduszkiewicz constant associated with the adsorption capacity (mg/g) β—the Dubinin–Raduszkiewicz constant related to the adsorption mean free energy (mol^2^/kJ^2^) E_a_—the activation energy (kJ/mol)
Sips	$q_{e}=\frac{q_{m}{\left(K_{}C_{e}\right)}^{n}}{{\left(1+K_{}C_{e}\right)}^{n}}$	q_m_, K—model’s constants (mg/g) and (L/mg) n—the heterogeneity index whose magnitude increases with heterogeneity

**Table 3:** Equilibrium capacities of Ni(II) sorption on different ion exchangers as follows from the literature reports.

Ion Exchangers	Sorption Conditions	Equilibrium Capacity (mg/g)	References
Amberlite IRA458	C_0_ 58.7 mg/L, t 240 min, T 293 K, in aqueous solution (Ni(II)-IDS 1-1)	5.89	[26]
Amberlite IRA958		5.84	
Amberlite IRA67		5.73	
Purolite S984	C_0_ 100 mg/L, t 240 min, T 298 K, in chloride solution (0.1 mol/L HCl)	4.95	[27]
Purolite A830		4.60	[28]
Lewatit MonoPlus SR7		4.56	
Purolite A400TL		4.72	
Dowex PSR2		3.70	[25]
Dowex PSR3		4.73	
Lewatit MonoPlus TP220		6.24	[29]
Lewatit AF5		4.89	[30]
Diphonix	C_0_ 100 mg/L, t 120 min, T 293 K, in nitrate solution (0.2 mol/L HNO_3_)	5.03	[31]
Amberjet^TM^ 1200H	C_0_ 2.44 mg/L, t 24 h, in real ammoniacal industrial wastewater	4.36	[32]
Purolite S940	C_0_ 2935 mg/L, t 7 days, 298 K, in chloride solution	4.22	[33]
Purolite S950		3.31	
Dowex PSR2	C_0_ 100 mg/L, t 240 min, T 293 K, in aqueous solution	10.30	this research

**Table 4:** Parameters of adsorption kinetics of nickel ions on Dowex PSR2 and PSR3 (C_0_ 100 mg/L).

Model		Parameters	Units	Ion Exchangers	
				PSR2	PSR3
PFO		q_e_	mg/g	10.30	19.10
		k_1_	1/min	0.028	0.024
		q_1_	mg/g	7.57	4.97
		R^2^	-	0.976	0.931
PSO		k_2_	(g/mg∙min)	0.008	0.023
		q_2_	(mg/g)	10.79	19.20
		R^2^	-	0.998	1.000
EKE		α	(mg/g∙min)	3.227	11,471.622
		β	(g/mg)	0.562	0.735
		R^2^	-	0.960	0.950
IPD	first step	k_i1_	mg/g/min^0.5^	1.534	1.988
		C_1_	-	0.168	9.916
		R^2^_1_	-	0.749	0.873
	second step	k_i2_	mg/g/min^0.5^	0.966	0.410
		C_2_	-	1.627	14.703
		R^2^_2_	-	0.864	0.821
	third step	k_i3_	mg/g/min^0.5^	0.060	0.061
		C_3_	-	9.374	18.152
		R^2^_3_	-	1.000	1.000

**Table 5:** Parameters of the isotherms for the nickel ions sorption on Dowex PSR2 and PSR3.

Model	Parameters	Units	Ion Exchangers	
			PSR2	PSR3
Langmuir	q_m_	mg/g	19.16	31.72
	K_L_	L/mg	0.012	0.021
	R^2^	-	0.972	0.995
Freundlich	K_F_	mg/g	1.15	2.00
	n	-	2.257	2.172
	R^2^	-	0.964	0.937
Temkin	A	L/g	0.257	0.376
	b_T_	kJ/mol	0.762	0.442
	R^2^	-	0.915	0.968
Dubinin-Raduszkiewicz	q_m_	mg/g	0.0005	0.0011
	β	mol^2^/kJ^2^	0.0049	0.0053
	E_a_	kJ/mol	10.074	9.753
	R^2^	-	0.945	0.969
	q_m_	mg/g	27.74	25.53
Sips	K	L/mg	0.002	0.0002
	n		0.634	0.469
	R^2^		0.982	0.989

**Table 6:** Thermodynamic parameters of the nickel ions sorption on Dowex PSR2 and PSR3.

Parameters	Temperature (K)	Ion Exchangers	
		PSR2	PSR3
K_d_	293	37.7	58.7
	313	34.3	52.5
	323	31.1	47.1
	333	28.9	43.7
ΔH° (kJ/mol)	-	−5.37	−6.01
ΔS° (kJ/mol)	-	11.96	13.47
ΔG° (kJ/mol)	293	−8.87	−9.96
	313	−9.11	−10.23
	323	−9.23	−10.36
	333	−9.35	−10.50

**Table 7:** XPS results obtained in a wide range of binding energy for the Dowex PSR2 and Dowex PSR3 ion exchangers before the Ni(II) ions sorption process.

Ion Exchanger	Name	Position (eV)	Raw Area	% Atom Concentration
Dowex PSR2	C 1s	284.7	23,366.6	83.3
	N 1s	401.7	618.9	2.2
	O 1s	532.2	3235.6	11.5
	Al 2p	74.7	139.3	0.5
	Si 2p	101.7	134.5	0.5
	S 2p	167.7	106.8	0.4
	Cl 2p	197.0	459.8	1.6
Dowex PSR3	C 1s	284.7	20,357.8	87.7
	N 1s	401.7	649.0	1.6
	O 1s	531.5	5529.7	8.1
	Cl 2p	197.0	1420.3	2.7

**Table 8:** XPS results obtained in a narrow range of binding energy for Dowex PSR2 and Dowex PSR3 ion exchangers before the Ni(II) ions sorption process [53,54,55].

Element	Position (eV)	Raw Area	% Atom Concentration	Phase
Dowex PSR2				
C 1s	284.7	3338.9	74.7	C=C, C-H, C-C
	285.9	1086.3	24.3	-CH-, CN
	288.6	45.3	1	C-H–Ar
O 1s	531.9	404.9	52.1	C-O
	532.9	372.3	47.9	nitrates
Cl 2p	196.8	68.3	50.7	Cl-
	198.4	66.5	49.3	–
N 1s	399.90	33.1	31.5	quaternary amine
	402.13	65.0	61.9	protonated amine
	406.14	7.0	6.6	nitro group
Dowex PSR3				
C 1s	284.7	7633.66	86.5	C=C, C-H, C-C
	286.2	1119.66	12.7	-CH-, CN
	288.7	74.7348	0.8	C-H–Ar
O 1s	532.2	2210.1	84.4	C-O
	533.8	408.3	15.6	nitrates
Cl 2p	196.6	264.8	74.4	Cl-
	197.9	132.4	-	-
	198.6	91.2	25.6	chlorates
	199.7	45.6	-	-
N 1s	401.9	374.6	100	quaternary amine

**Table 9:** XPS results obtained in a wide range of binding energy for Dowex PSR2 and Dowex PSR3 ion exchangers after the Ni(II) ions sorption process.

Ion Exchanger	Name	Position (eV)	Raw Area	% Atom Concentration
Dowex PSR2	C 1s	284.7	23,842.4	80.1
	N 1s	401.7	889.7	3.0
	O 1s	532.2	4281.0	14.4
	Al 2p	73.2	277.1	0.9
	Si 2p	101.7	363.1	1.2
	S 2p	167.7	48.8	0.2
	Cl 2p	197.0	81.4	0.3
Dowex PSR3	C 1s	284.7	18,315.3	82.3
	N 1s	401.7	1249.1	3.1
	O 1s	532.2	9236.3	14.2
	Cl 2p	197.7	235.3	0.5
	Ni 2p	856.2	482.5	-

**Table 10:** XPS results obtained in a narrow range of binding energy for Dowex PSR2 and Dowex PSR3 ion exchangers after the Ni(II) ions sorption process [53,54,55].

Element	Position (eV)	Raw Area	% Atom Concentration	Phase
Dowex PSR2				
C 1s	C 1s A	284.7	77.5	C=C, C-H, C-C
	C 1s B	286.0	21.4	-CH-, CN
	C 1s C	288.7	1.1	C-H–Ar
O 1s	O 1s A	533.9	5.2	C-O
	O 1s B	532.2	94.8	nitrates
Cl 2p	Cl 2p 3/2	11.4	50.7	Cl-
	Cl 2p 1/2	11.1	49.3	–
N 1s	N 1s A	399.8	21.2	quaternary amine
	N 1s B	402.2	50.7	protonated amine
	N 1s C	406.1	28.1	nitro group
Dowex PSR3				
C 1s	284.7	5194.3	63.6	C=C, C-H, C-C
	284.9	1122.9	13.7	-CH-, CN
	286.1	1378.5	16.9	C-H–Ar
	288.7	457.8	5.6	-COOH
	291.2	18.9	0.2	π→π *
O 1s	531.9	2817.6	63.5	C-O
	533.4	1621.2	36.5	nitrates
Cl 2p	196.8	114.2	100.0	Cl-
	199.5	57.1	-	-
N 1s	401.9	239.7	66.1	quaternary amine
	405.9	123.0	33.9	nitro group

* transition from ground to excited state

**Table 11:** Percentage of elements in the Dowex PSR2 and Dowex PSR3 ion exchangers before the Ni(II) ions sorption process.

Ion Exchanger	Element	Measurement I [%]	Measurement II [%]	Mean (%)
Dowex PSR2	C	70.08	70.22	70.15
	H	8.81	8.79	8.80
	N	2.80	2.77	2.78
Dowex PSR3	C	74.81	74.73	74.77
	H	12.07	12.10	12.09
	N	2.41	2.41	2.41

**Table 12:** Elemental composition of the Dowex PSR2 and Dowex PSR3 ion exchangers was determined using the CHN method and the theoretical calculations prior to the Ni(II) ions sorption process.

Ion Exchanger	Element	% Content Determined by the CHN Method	Theoretical% Content (1:1) 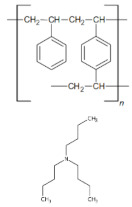	Theoretical% Content (2:1) 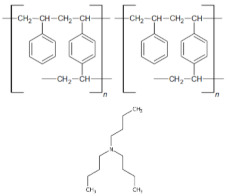	Theoretical% Content (1:2) 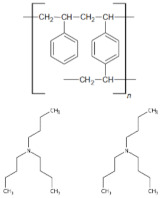
Dowex PSR2	C	70.15	86.12	88.34	83.72
	H	8.80	10.53	9.51	11.63
	N	2.78	3.35	2.15	4.65
Dowex PSR3	C	74.77	86.12	88.34	83.72
	H	12.09	10.53	9.51	11.63
	N	2.41	3.35	2.15	4.65

## Data Availability

Data are contained in the paper and Supplementary Material.

## Supplementary Materials

The following supporting information can be downloaded at: https://www.mdpi.com/article/10.3390/ma16020644/s1, Table S1: Parameters of adsorption kinetics of nickel ions on Dowex PSR2 and PSR3 (C_0_ 10–50 mg/L). Figure S1: XPS spectra of carbon, oxygen, nitrogen, and chlorine made for the Dowex PSR 2 ion exchanger before the sorption process. Figure S2: XPS spectra of carbon, oxygen, nitrogen, and chlorine made for the Dowex PSR 2 ion exchanger after the sorption process. Figure S3: XPS spectra of carbon, oxygen, nitrogen, and chlorine made for the Dowex PSR 3 ion exchanger before the sorption process. Figure S4: XPS spectra of carbon, oxygen, nitrogen, and chlorine made for the Dowex PSR 3 ion exchanger after the sorption process.
