# Supplementary material for: Determination of the Ni(II) Ions Sorption Mechanism on Dowex PSR2 and Dowex PSR3 Ion Exchangers Based on Spectroscopic Studies
Source: Materials (Basel). 2023 Jan 9;16(2):644. doi: 10.3390/ma16020644 (PMC9866840; doi:10.3390/ma16020644)
Supplement: Supplementary file 1 [file materials-16-00644-s001.zip › materials-2127337-supplementary.pdf]

materials

MDPI

Supplementary Material

Determination of the Ni(II) Ions Sorption Mechanism on Dowex PSR2 and Dowex PSR3 Ion Exchangers Based on the Spectroscopic Studies

Justyna Bąk[1,*], Weronika Sofińska-Chmiel[2], Maria Gajewska[2], Paulina Malinowska[2] and Dorota Kołodyńska[1]

[1] Department of Inorganic Chemistry, Institute of Chemical Sciences, Faculty of Chemistry, Maria Curie-Skłodowska University, Maria Curie-Skłodowska Sq. 2, 20-031, Lublin, Poland
[2] Analytical Laboratory, Institute of Chemical Sciences, Faculty of Chemistry, Maria Curie Skłodowska University, Maria Curie Skłodowska Sq. 3, 20-031 Lublin, Poland.
* Correspondence: justyna.bak@mail.umcs.pl

Table S1. Parameters of adsorption kinetic of nickel ions on Dowex PSR2 and PSR3 (C_0 10-50 mg/L).

Model		Parameters	Units	Ion exchangers					
				PSR2			PSR3		
		Concentration	mg/L	10	25	50	10	25	50
		q_e	mg/g	2.24	3.30	4.92	2.51	6.76	8.59
PFO		k_1	1/min	0.026	0.025	0.028	0.020	0.020	0.018
		q_1	mg/g	1.85	1.83	1.64	1.13	1.21	2.13
		R^2		0.971	0.945	0.859	0.975	0.969	0.770
PSO		k_2	(g/mg·min)	0.019	0.043	0.068	0.070	0.089	0.049
		q_2	(mg/g)	2.46	3.38	4.97	2.54	6.78	8.61
		R^2		0.995	0.999	1.000	0.998	1.000	1.000
EKE		α	(mg/g·min)	0.358	2.477	16.870	18.104	17338.402	1745.721
		β	(g/mg)	2.190	2.005	1.584	3.885	3.445	1.495
		R^2		0.950	0.928	0.962	0.973	0.989	0.910
Webber-Morris	First step	k_{i1}	mg/g/min$^{0.5}$	0.438	0.700	1.400	0.289	0.231	1.039
		C		-0.462	-0.012	-0.001	0.852	5.080	3.939
		R^2		0.798	0.817	0.868	0.912	0.964	0.993
	Second step	k_{i2}	mg/g/min$^{0.5}$	0.179	0.199	0.225	0.078	0.139	0.298
		C		0.572	1.429	3.211	1.446	5.396	6.232
		R^2		0.976	0.929	0.968	0.982	0.948	0.993
	Third step	k_{i3}	mg/g/min$^{0.5}$	0.018	0.061	0.017	0.024	0.052	0.073
		C		1.956	2.410	4.647	2.150	5.990	7.460
		R^2		1.000	0.870	1.000	1.000	0.945	1.000

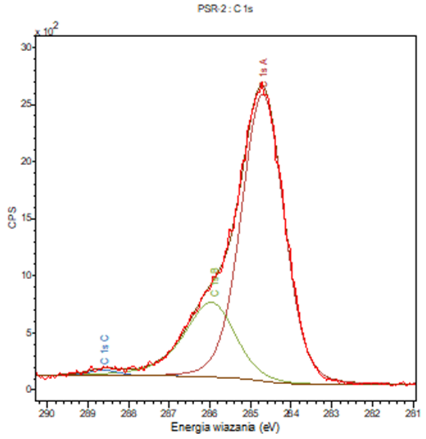

(**a**)

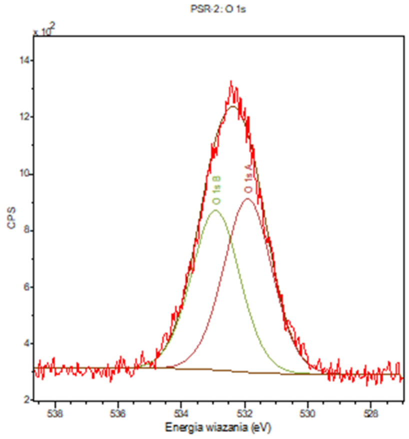

(**b**)

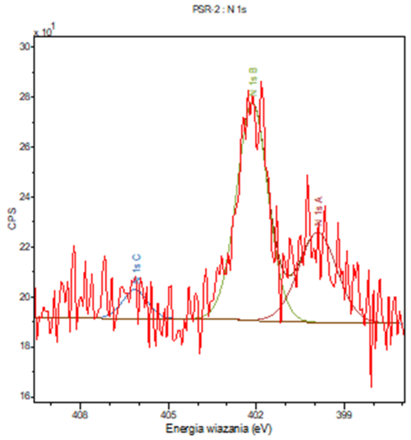

(**c**)

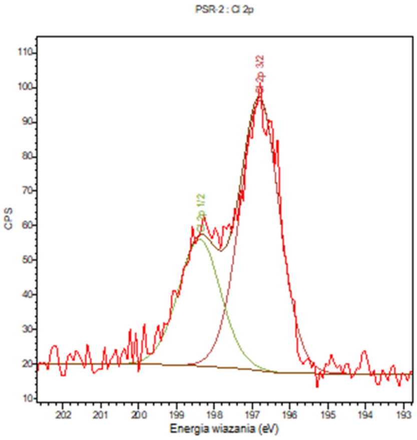

(**d**)

Figure S1. XPS spectra of carbon, oxygen, nitrogen and chlorine made for the Dowex PSR 2 ion exchanger before the sorption process.

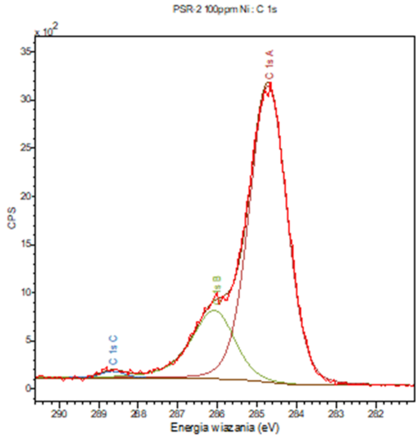

(**a**)

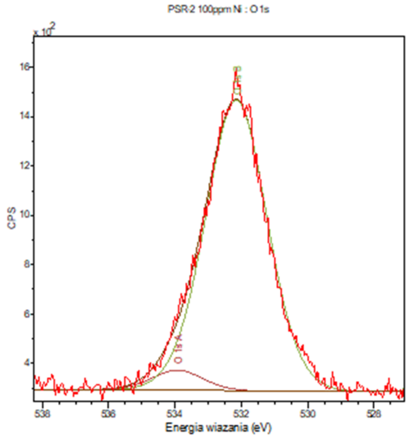

(**b**)

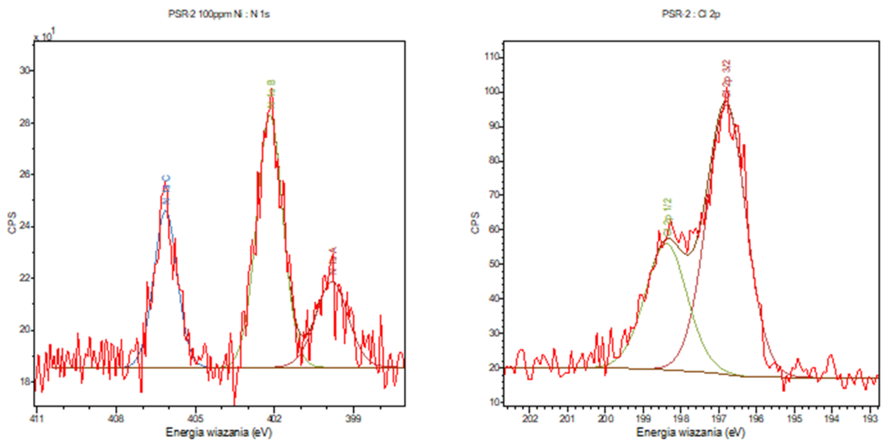

(**c**) (**d**)

Figure S2. XPS spectra of carbon, oxygen, nitrogen and chlorine made for the Dowex PSR 2 ion exchanger after the sorption process.

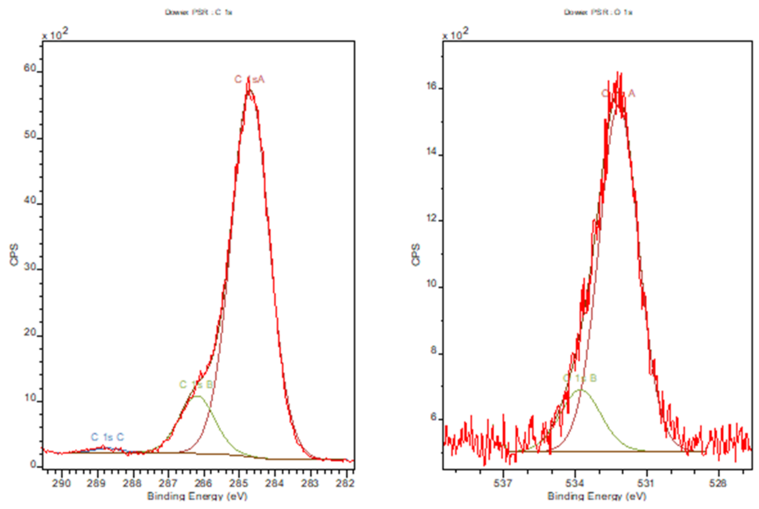

(**a**) (**b**)

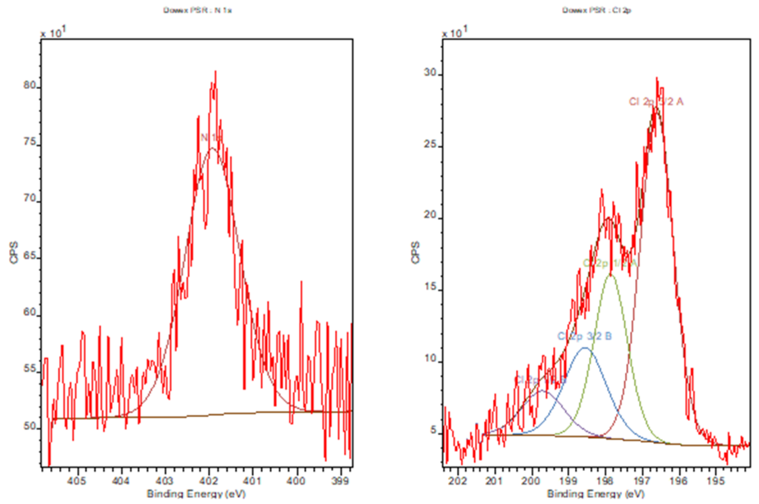

(**c**) (**d**)

Figure S3. XPS spectra of carbon, oxygen, nitrogen and chlorine made for the Dowex PSR 3 ion exchanger before the sorption process.

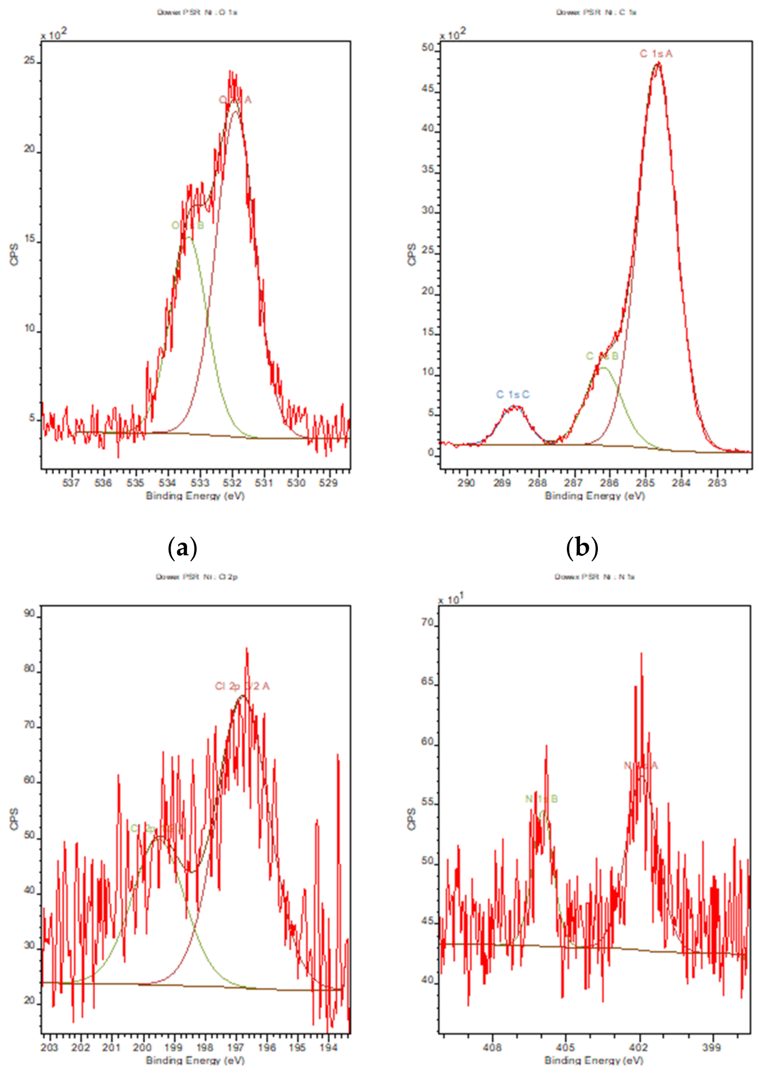

(**a**) (**b**) (**c**) (**d**)

Figure S4. XPS spectra of carbon, oxygen, nitrogen and chlorine made for the Dowex PSR 3 ion exchanger after the sorption process.
